# Lines in the sand: pre-interview rank and probability of receiving admission to medical school

**Published:** 2019-07-24

**Authors:** Raquel Burgess, Meredith Vanstone, Margo Mountjoy, Lawrence Grierson

**Affiliations:** 1 Department of Family Medicine, McMaster University, Ontario, Canada; 2 Undergraduate Medical Education Program, Michael G. DeGroote School of Medicine, McMaster University, Ontario, Canada; 3 McMaster Education Research, Innovation and Theory (MERIT) program, Faculty of Health Sciences, McMaster University, Ontario, Canada

## Abstract

**Background:**

We provide an examination of one medical school’s attempt to determine whether their cut-off point for number of interviews offered is congruent with the probability these applicants’ have for admission post- interview.

**Methods:**

Offer probability was determined by organizing pre-interview rankings from 2013-2017 (n = 2,659) applicant cohorts into bins of 50 applicants and finding the quotient of successful and total applicants in each bin. A linear-by-linear association Chi-square test and adjusted standardized residuals with an applied Bonferroni correction were used to determine if the observed frequencies in each bin were different than expected by chance. A Spearman Correlation analysis between pre- and post-interview ranks was conducted.

**Results:**

All applicants have between a 50.0% and 76.4% chance of admission. Observed frequencies are different than chance (χ(1)=50.835, p<.001), with a significantly greater number of offers seen in the bins between 1 and 100 (p<.001 for both bins). There is a weak positive relationship between pre- and post-rank, r_s_(2657)= 0.258, p<.001.

**Conclusion:**

The results indicate the number of interviews conducted does not exceed a threshold wherein individuals with a relatively low chance of admission are interviewed. Findings are interpreted with respect to ethical resource allocation for both programs and applicants.

## Introduction

The critical importance of admissions procedures to medical school becomes clear when one considers the relative power of selection factors compared to educational interventions on gains in performance in medical school.^1^ Indeed, there is a prolific amount of research dedicated to ensuring that admissions procedures select for particular traits,^2,3^ that they assess applicants holistically and comprehensively,^4–6^ and that they adequately predict future performance as a physician.^7–10^ Importantly, medical schools receive an enormous number of applications,^9^ necessitating the creation of staged admissions procedures in order to assess the applicants in an effective and efficient manner. An inherent feature of these procedures is the use of pre-established thresholds that serve as the criterion for determining the candidates that continue forward to more intensive means of assessment. For example, it is typical for schools to set minimum grade point averages (GPA) to select applicants to participate in an interview. The determination of these thresholds is oriented toward balancing effectiveness and efficient distribution of resources and the impacts that these decisions have on the characteristics of the applicant pool.

At McMaster University in Hamilton, Canada, the medical school uses a staged application process for selecting students. Stage 1 ranks the applicants as a function of their cumulative undergraduate GPA (32%), MCAT Verbal Reasoning (VR) or Critical Analysis and Reasoning (CARS) score (32%), and CASPer score (32%), with an additional bonus awarded to those with a Masters (1%) or PhD (4%) degree. From there, approximately 550 applicants proceed to Stage 2, which involves participation in a Multiple-Mini Interview (MMI).^11^ Offers of admission to medical school are sent to approximately the top 203 ranked candidates according to a second algorithm consisting of 70% MMI score, 15% GPA, and 15% MCAT VR/CARS. In the context of budget constraints and the understanding that resources allocated to one domain of the medical school process (e.g., selection procedures) inevitably divert resources from other domains (e.g., curriculum enhancement), the school was interested in understanding whether the interview process was making appropriate use of the available resources (i.e., *should McMaster’s medical school interview fewer applicants?*). Interviewing 550 applicants requires three days of time involving 18 managerial staff, three faculty directors, 80 assessors/day, eight standardized actors/day, and 36 student hosts. The MMI process costs the program approximately $75 per applicant, which accounts for the costs of catering, parking, and staff (assessors, actors and student hosts are volunteers). There are additional costs associated with station creation, correspondence, and the score review process. Moreover, the potential for resource burden also extends to the applicants. Interviews for McMaster’s medical program are conducted in-person on a specific date with little exception, and the costs of travel and accommodation to participate falls to the applicant.

Therefore, the purpose of this research study was to examine how the probability of receiving an offer varies based on the applicants’ pre-interview rank, and to understand better the relationship between pre- and post-interview ranks. To answer this question, our research team collected several years of data pertaining to the staged application process at McMaster University and constructed statistical models to investigate these relationships. We believe this provides a useful method of evaluating the utility of the threshold our medical school set for interviews. If we were to demonstrate a substantial and statistically significant drop in the likelihood that applicants below a particular pre-interview rank threshold receive an offer of admission, then the medical school would consider inviting fewer applicants to interview.

## Methods

### Institutional approval

This study received an ethics waiver from the Hamilton Integrated Research Ethics Board (HIREB) according to provisions for research pertaining to programmatic evaluation and quality improvement involving locally accessible data sources. As the data in question is de-identified, HIREB also waived the requirement for written informed consent.

### Data

Data pertaining to 2,659 applicants to the McMaster University Undergraduate Medical Education Program between 2013 and 2017 were collated. Data included pre-interview rank, post-interview rank, and an indication of whether the applicant had received an offer or been refused for all 2,659 applicants. For the purposes of this study, “offer” refers to offers accepted, offers tendered and declined, and offers following wait-listing. The indication of “refused” includes offers that were not tendered regardless of wait-listed status.

### Analyses

All analyses were conducted using SPSS Version 25.0.^12^ The chance probability of an interviewing applicant receiving an offer was calculated by dividing the number of tendered offers by the number of applicants, and multiplying the resulting coefficient by 100%. A Cochran-Armitage test of trend was conducted to determine the linear relationship between the pre-interview ranks and offer status.

A linear-by-linear association Chi-square test was performed.^13^ This involved grouping applicants into bins of 50 (i.e., 11 bins/cohort) and determining whether the observed frequency of offers across bins was significantly different than expected by chance.

Post-hoc tests using adjusted standardized residuals were used to ascertain the specific bins in which significant differences were present.^14^ This involved squaring the *z*-scores to determine the associated Chi- square values, which were then used to determine the corresponding p-values using the Chi-square significance function in SPSS. A Bonferroni correction was applied to account for the calculations across the 11 bins. This resulted in the alpha-value of .05 for statistical significance being adjusted to .002. A conservative adjustment of the alpha-value was deemed appropriate given that a rejection of the null hypothesis could result in a consequential programmatic change (i.e., the medical program would consider offering less interviews to future applicant cohorts).

The quotient of the number of offers in each bin and the number of total applicants in each bin was calculated to determine the probabilities of receiving an offer as a function of pre-interview rank bin.

A Spearman Correlation analysis was used to determine the relationship between pre-interview rank and post-interview rank.

## Results

The chance probability of an interviewee receiving an offer was 58.7%. This corresponds to an average of 312 offers per cohort, which reflects the process of filling 200 spots by way of a rolling admissions procedure (i.e., extending offers to waitlisted candidates when primary tenders are declined).

The results of the Cochran-Armitage test of trend revealed a significant linear trend between pre- interview rank and offer status; p<.001. In particular, as pre-interview rank increased (i.e., a less favourable rank), the number of offers following the interview decreased.

The Chi-square test indicated that this trend includes observed frequencies of offers within bins that are significantly different than those expected by chance; χ(1)=50.835, p<.001. Specifically, post-hoc testing shows that a significantly greater number of offers are tendered to applicants categorized into the top bin (pre-interview ranks 1 to 50), *z* = 3.66 p<.001, and second bin (pre-interview ranks 51 to 100), *z* = 5.96 p<.001. Moreover, this testing revealed no significant differences amongst the remaining bins (i.e., comprising pre-interview ranks 101 to 550) (Table 1).

**Table 1:** Probability of receiving an offer based on pre-interview rank bin for McMaster University’s Undergraduate Medical Education Program 2013- 2017 applicant cohorts.

Bin	Pre-interview rank	Number of offers	Number of applicants	Offer probability (%)
1	1-50	174	250	69.6 *
2	51-100	191	250	76.4 *
3	101-150	160	250	64
4	151-200	158	250	63.2
5	201-250	131	250	52.4
6	251-300	133	250	53.2
7	301-350	127	250	50.8
8	351-400	145	250	58
9	401-450	135	250	54
10	451-500	125	250	50
11	501-550	83	159	52.2

* denotes a significantly different number of offers than expected based on an alpha value of p < .002 (applied Bonferroni correction).

The Spearman Correlation analysis revealed a weak positive relationship between pre-interview and post- interview ranking, r_s_(2657)= 0.258, p<.001. This indicates that the pre-interview rank is not a strong predictor of whether or not an applicant receives an offer.

## Discussion

This study sought to determine the relationship between applicant pre-interview rank and likelihood of receiving an offer of admission into the Undergraduate Medical Education Program at McMaster University, asking whether there was a significant inflection point in pre-interview rank after which applicants were unlikely to receive an offer. In this regard, the findings of the analysis indicate that there is a significant linear trend between pre- interview rank and offer status, with all applicants having between a 50.0% and 76.4% probability of receiving an offer. Interestingly, the analyses reveal that applicants ranked in the top 100 of the candidate pool have a statistically significantly higher chance of receiving an offer than those ranked 101-550. However, applicants receiving a ranking of 201 or more still experience a 50.0% or greater chance of receiving an offer of admission. Given that this significant inflection point in the linear trend is associated with a ranking that would include fewer applicants than available spots, we can conclude that the cut-off criterion of 550 interview spots does not seem to be a misallocation of resources; that is, we are not using resources to interview applicants that have a poor chance of admission. Indeed, the data may be interpreted in a way that suggests that the McMaster medical school is interviewing too few applicants, and that the interview determination criterion may be excluding suitably-qualified applicants with below threshold pre-interview ranks from receiving a holistic appraisal.^4–6^

Taken together, these data require that the McMaster medical school consider the possibility that an arbitrary “*line-in-the-sand*” has been drawn to balance resource constraints and comprehensiveness. Every institution is required to do this, and it is important to consider the implications of where this line is drawn. Other institutions may consider conducting similar analyses specific to their institutional processes to ascertain the implications that the line they have drawn has for their available resources. In our institution, these data challenge us to consider the possibilities of interviewing additional candidates and the associated opportunity cost: additional resources spent on selection procedures could otherwise be allocated to the educational activities that eventually support enrolled and active students. In this regard, questions of *worth* are raised. Does improved selection reduce costs elsewhere; for example, those associated with subsequent underperformance, professionalism issues, and/or remediation processes? The relatively higher potential of selection factors on performance gains in medical school when compared to educational interventions^1^ supports this line of thinking.

Furthermore, it is important we recognize that judgements of “low” or “high” probability of admission have a subjective quality. That is, we must be careful not to conflate programmatic determinations of “*worth”* with what probabilities are “*worth it*” for applicants. After all, decisions about applications are similar to other decisions with regards to the need to balance the probability of success against one’s associated effort and available resources. In the case of interviews to medical school, personal and financial resources required of applicants to attend the interview may range from transportation costs to time-off paid employment to money spent on professional interview attire. Some applicants may be in a position to afford these costs for only one interview at one institution, whereas others may be able to afford multiple interview attempts. It is not within our jurisdiction to decide what is “*fair*” and “*worth it*” for each individual applicant based on probability analyses alone. One potentially constructive recommendation resulting from this analysis is to ask whether applicants should be afforded the opportunity to know their pre- interview rank and the associated probability of admission when being invited to interview. This would allow applicants to come to their own conclusion as to whether or not the interview is worth the time, energy, and financial cost. With respect to the line(s) that institutions are required to create within their admissions processes, further research should seek to investigate how selection formulae influence where the line is drawn and how this impacts the applicant pool. Moreover, further investigation could lend greater insight into the ways in which the selection of admissions thresholds can most effectively balance resource and opportunity cost considerations, feasibility, and the holistic appraisal of suitably-qualified applicants.
